# The Rich

**Published:** 1871-05

**Authors:** 


### HALL'S

### JOURNAL OF HEALTH.

VOL. XVIII. — MAY, 1871. — NO. V.

### THE RICH.

EVERYBODY seems to have a spite against rich people; unfavorable constructions are placed on their conduct, their motives are impugned, and they are regarded as the common enemies of mankind. The news that a rich man has fallen into misfortune, has lost a large amount of money, or suffered disgrace himself or through some member of his family, gives a chuckle of delight, a secret sense of satisfaction. These unworthy feelings arise in part from one of the meanest of all human traits, envy; and in other part, because the rich do not use their money as we think they ought to do. The secular newspapers do a great deal towards fostering these feelings. The following is going the rounds of the press at this time, as coming from Newport: "It is a well known fact that a wealthy New Yorker, who owns a superb residence here, every spring removes the thirty-seven cents tassels from his curtains in New York, brings them on, attaches them to his curtains here, and in the fall takes them back to town with him."

Suppose the statement is true, the presumption is the tassels were paid for, and rightfully belong to the person alluded to. He bought them for use: will you dictate to him what he shall do with his own property? Does a man lose the absolute right over his own, because he is rich? Rich people are rich, and remain so through life, simply because they take care of their money; because they practice daily self-denials and avoid extravagant expenditure and waste, from principle, habit, and inclination. They rightly think that waste is wicked; they have cultivated the habit of saving; that habit has become a pleasure, and it does them more good to save than to squander; and when a man has spent half a century in the industrious and honorable effort to make money in a legal manner, shall he not be allowed to use it in the way which affords him the greatest pleasure? The man who earns and spends one dollar to suit himself, does not lose his right when that one has become a million.

And when we take into account that as we get older, we get weaker, less able to work, less heart to work, are more and more left alone in the world, by the friends of our youth dying, and who would have helped us had we fallen into extremity; and when we remember, too, that with age comes sickness and pain and helplessness, and that money will always purchase attentions, and will supply our wants; and over all this, when we know that the world is selfish, and that human friendship fails, it ought not to be set down as a crime, that with increasing need of help from others, we should grow increasingly careful of that money which will always and with certainty command that attention and that help.

### "HE CAN AFFORD IT."

Mr. Stewart is often complained of and very bitterly too, that he pays poor salaries to those who work for him. If you go through his establishment, and catechise each of the thousand whom he employs, three out of four may complain that he does not pay them enough. And very sensible people often take up this complaint. If you question these dissatisfied persons closely, as to the grounds of their discontent, it will come out in the expression, "He is rich, and would never miss it if he were to pay us liberal salaries." But every reader, rich or poor, is personally interested in looking at the other side of the question.

Mr. Stewart pays his young men as much as he thinks their services are worth to him. Who of all our readers is willing to pay more for work than it is worth?

These dissatisfied persons are not compelled to remain; the very fact of their remaining shows that Mr. Stewart pays them at least as much as any one else is willing to pay them for the work they do. Mr. Stewart is too shrewd a business man not to see the worth of those employed by him, and no doubt promotes to higher salaries those who deserve it; it is to his business interest to do so.

### THE PUBLIC GOOD.

A young man who will habitually receive more for his services than they are worth, takes the first steps towards becoming a dishonest man, because he is learning the habit of taking what he does not earn, and to that extent becomes willing to receive a gift, a something for which he has not worked, for which he has made no compensation, and to that extent loses his own self-respect, his own manliness, and thereby shows his own unworthiness.

If Mr. Stewart pays his young men more than they are worth, then others, whose profits will not warrant it, must pay more than those they employ are worth; in this way a great hardship would be imposed on the business community at large, and one of two results would necessarily follow: tradesmen must either fail, or refuse to employ those who demand more than their services are worth, and would attempt to do more themselves; then those who wanted exorbitant wages would not be employed at all, would have nothing to do; hence must be supported by their friends or relations, or fall into sharp practices, downright roguery, and finally reach the penitentiary. Hence, the men who pay moderate salaries, although of immense wealth, are doing the public a substantial good, in simply paying for services what those services are worth, and no more. Besides, who supports the government? The rich men who pay taxes. Whose money fills our public libraries, endows our colleges, our theological seminaries, and our institutes; builds asylums for the hapless insane, "Homes" for helpless age, for pitiful infancy, for the unfortunate poor and sick and demented? It is the money of such rich men as Daniel Drew, A. T. Stewart, Ezra Cornell, Peter Cooper, George Peabody, Orange Judd, Robert Bonner, James Lenox, and the thousand noble-hearted men like them; not forgetting Alexander and Robert L. Stuart, whose large benevolences have been habitual for a score of years or more; and then there is Jay Cooke, with his large heart, who "has just given his annual picnic to his Bible-class, near Philadelphia, where he has erected at his own expense a neat school-house, a pretty chapel, and a commodious hall which he built long ago for the religious instruction of working men on his place, himself being their teacher, the class numbering a hundred and fifty members. Then come all sorts of athletic games and amusing employments, ending with a sumptuous dinner and ice-cream and music, himself as happy as the happiest." And, later on, we suppose he will go to his "island in the lake," and take with him a young regiment of clergymen of all creeds, as is his wont, to be his guests in the heats of summer, where, without expense, they may be entertained and recreated for their own benefit and that of the church to whose interests they have consecrated their time, their talents, their hearts, and their lives.

The fact is, if a man has a talent for making money, it is his highest duty to make all he can, and to save it, so as to have the means of extending his operations and make thousands where he is now making but hundreds; so that in the end he may give millions to humanity instead of tens. Rich men deserve to be honored; and although in our little pitiful envy we may be forever pecking at them, and expending upon them our little spiteful remarks and our spleen, there is one thing certain, they command our respect, and we can't help but look up to them with a certain degree of homage and deference.

A poor little boy once found it very hard to get a few books to read, and the feeling came across his young and generous heart, that it would do him a great deal of good to be able, some of these times, to help boys to an easier way of improving themselves than had fallen to his lot. Had he given away every dollar he could spare, he would never have been able to give the larger part of a million of dollars to help the young to get an education without expense, as that grand old gentleman has done whom every New Yorker calls with pride, "PETER COOPER."

A poor German youth made a little money, and saved it. He saw that all the inhabitants of the village had to go a great way every day, and lose precious time in bringing water to town. The young man thought about it a great deal; still went on in his business, made no show, dressed plain, lived economically and saved money all the time; meanwhile he got to be an old, gray-headed man; no one seemed to care much about him, because he was "too close." One day he died; it was soon found that he had left a large amount of money, and a will directing that every dollar of it should be expended in works to bring abundant water to his native village, and to keep them in repair forever. That man's whole life was a heroism.

He had splendid prospects,—family, education, wealth; but by a calamity he became lame for life. In the course of years he was able to walk on crutches; and hours and hours in the long years, he stumped across the floor in his garret for exercise, and became at last to be thought a close-fisted, mean old fellow. The older he got the richer he grew, and the more he thought and planned and devised, until one day he died; and when his will was found, it was discovered that he had left a million of dollars to build and support a hospital for the halt and lame; and the name of James H. Rosevelt will be revered by the unfortunate crippled for all time.

As a very general rule in this country, the men who are rich have become so by long years of industry, economy, and self-denials; and that they remain rich, is owing to the fact of their continuance in the practice of these duties; duties to themselves, to those dependent on them, and to society in general, by which they are enabled in the end to give their thousands, their tens of thousands, and their millions to the foundation of institutions which are to feed the poor, to clothe the naked, to befriend the friendless, to aid the unfortunate, to nurse the sick, and give help and homes to the homeless and the old.

Col. T. was my next-door neighbor. He worked six years under a shed in New York city, before he felt able to give more than a shilling for his dinner. One day he went to three different places to get a head of lettuce in the early spring for a cent or two less than was asked, because he thought the price was too exorbitant; the very next day he gave ten thousand dollars to equip a regiment for the war. He was economical from habit and from principle, and both compelled him to be so until he died, leaving over a million of dollars, having built and endowed an academy for the education of poor children in his native town, the very town from which he used to walk six miles to the jail every Saturday afternoon, to take his father a clean shirt, — a father who had been a prosperous and wealthy merchant, but, having failed in business, was incarcerated for debt in the barbarous times of the past. Multitudes of similar instances could be given in illustration of the economy, the self-denials, and the princely liberality of our rich men; hence honor be to them everywhere.

